# Thyroid Abscess

**DOI:** 10.1210/jcemcr/luae056

**Published:** 2024-04-15

**Authors:** Iram Hussain, Alexander H Tessnow

**Affiliations:** Division of Endocrinology, Department of Internal Medicine, University of Texas Southwestern Medical Center, Dallas, TX 75390-8537, USA; Division of Endocrinology, Department of Internal Medicine, University of Texas Southwestern Medical Center, Dallas, TX 75390-8537, USA

**Keywords:** thyroid abscess, thyroid nodule

## Image Legend

**Figure 1.**
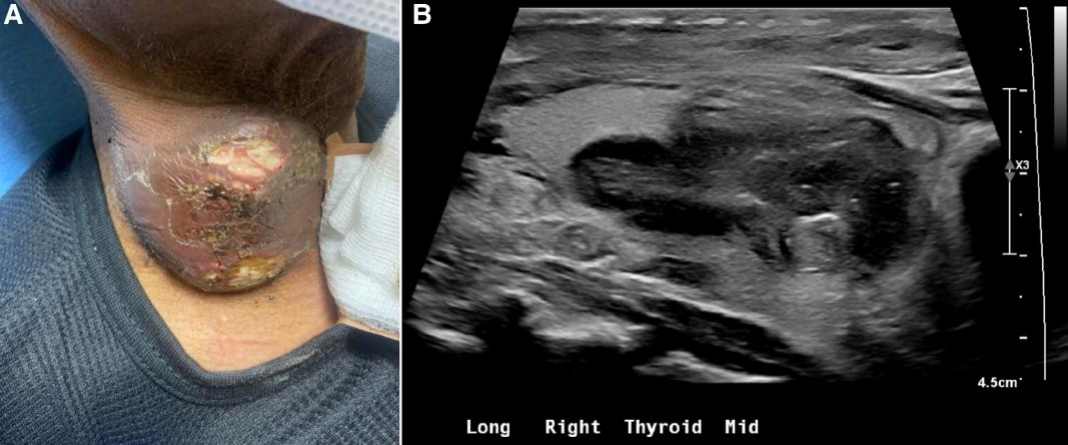
Thyroid abscess with overlying ulcerated skin and purulent drainage protruding from the anterior surface of the patient's neck (A) along with corresponding ultrasound appearance seen in sagittal view (B).

A 61-year-old man presented with a 7-day history of purulent discharge and skin ulceration over a thyroid nodule (Fig. 1A). The nodule was identified one year previously as complex/cystic (dimensions unknown) and biopsied with benign cytology. Two months prior to presentation, he was diagnosed with immune complex-mediated glomerulonephritis. Computed tomography imaging confirmed pneumonia and a right thyroid nodule, noted to be suspicious on ultrasound neck (Fig. 1B). He received ceftriaxone/azithromycin with clinical improvement followed by 2 infusions of rituximab. He was referred for outpatient thyroid evaluation.

He was afebrile (36.8 °C) with a normal white blood cell count and serum thyrotropin. The nodule was nontender and measured 44 mm × 27 mm × 37 mm. Repeat ultrasound-guided biopsy showed purulent fluid with atypia of underdetermined significance and culture revealed Klebsiella pneumoniae. The abscess was treated with ceftriaxone and metronidazole followed by amoxicillin. It reduced significantly in size and the drainage stopped. As the pneumonia was treated before rituximab, the thyroid was likely seeded with bacteria prior to immunosuppression. Membranous nephropathy can occur with infections as in this case of pneumonia and thyroid abscess [1]. This case highlights the importance of excluding infection, even in unusual sites such as the thyroid, prior to initiating rituximab.
